# Plant-based secondary metabolites as natural remedies: a comprehensive review on terpenes and their therapeutic applications

**DOI:** 10.3389/fphar.2025.1587215

**Published:** 2025-05-19

**Authors:** Sheeba Khanam, Pooja Mishra, Tabrez Faruqui, Pravej Alam, Thamer Albalawi, Faiza Siddiqui, Zeeshan Rafi, Salman Khan

**Affiliations:** ^1^ Department of Biosciences, Integral University, Lucknow, Uttar Pradesh, India; ^2^ Department of Personalized and Molecular Medicine, Era University, Lucknow, India; ^3^ Department of Biology, College of Science and Humanities, Prince Sattam bin Abdulaziz University, Al-Kharj, Saudi Arabia; ^4^ Department of Bioengineering, Integral University, Lucknow, Uttar Pradesh, India

**Keywords:** antibiotics, tumor microenvironment, cancer, natural compounds, plant-based therapy, terpenes

## Abstract

Terpenes are among the most diverse kinds of natural products because of their remarkable chemical variety. Numerous biological characteristics of terpenoids have been documented, including their antibacterial, antifungal, antiviral, antihyperglycemic, anti-inflammatory, and antiparasitic effects, as well as their cancer chemopreventive benefits. Additionally, terpenes are utilized in the manufacturing of organic solvents, varnishes, inks, adhesives, synthetic polymers, natural rubbers, cleaning supplies, biofuels, insecticides, and food and beverage items. Terpenes are therefore highly valued in modern medicine, pharmacy, nutraceuticals, cosmetics, and other fields. Plant oils, including terpenes, have been used to treat a variety of diseases without a full understanding of the roles or modes of action of particular bioactive substances. Many of these compounds are only present in nature in extremely small amounts; thus, methods such as metabolic engineering and synthetic biology are used to harvest them in large quantities in order to produce enough medicine. This comprehensive review aims to elucidate the biochemistry, phytochemical properties, and pharmacological activities of terpenes in metabolic disorders.

## 1 Terpene: biochemical and phytochemical properties

**FIGURE 1:**
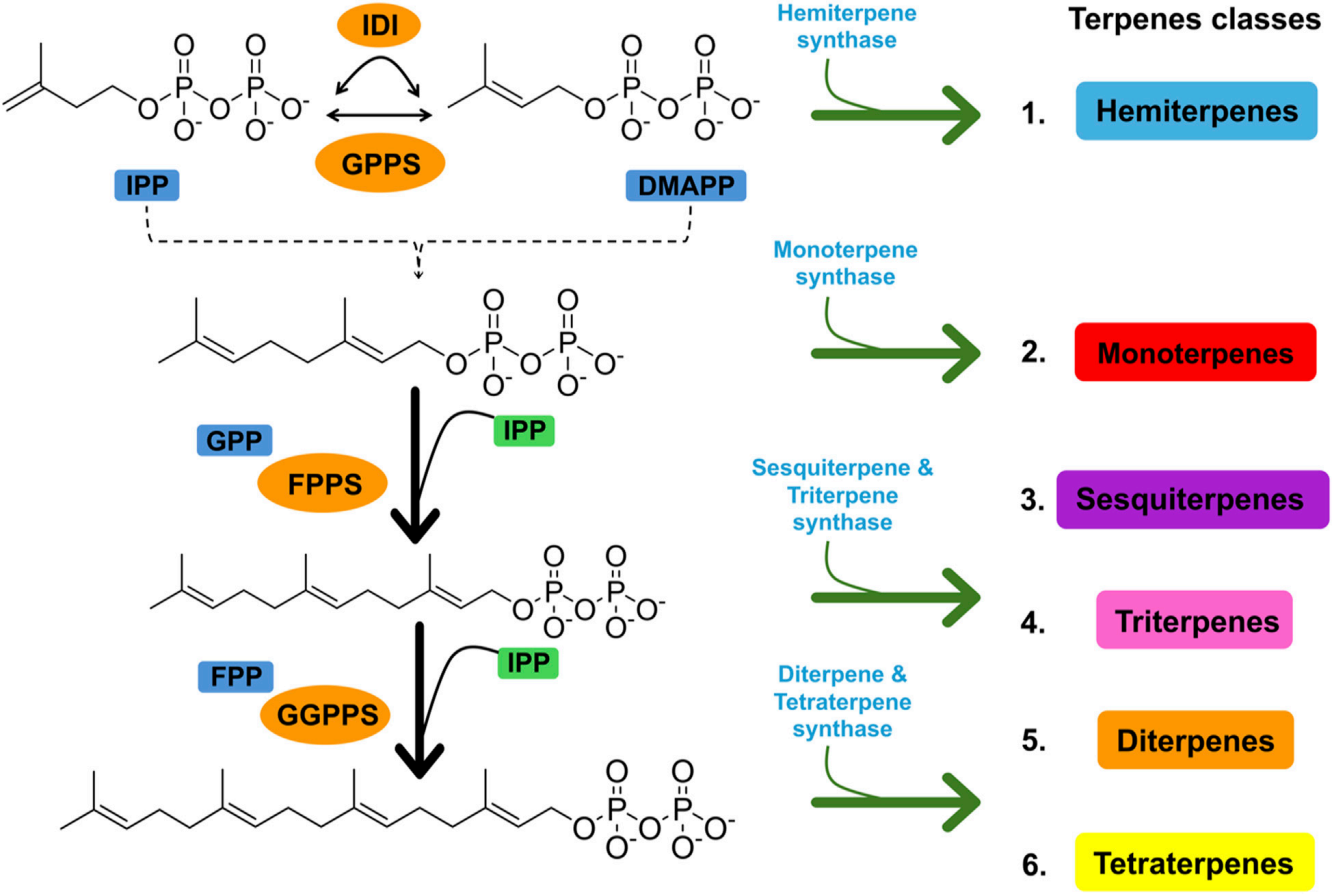
Figure showing the synthesis of different terpenes using two precursor molecules, the IPP unit and its isomer, DMAPP.

Terpenes are the largest and most functional class of secondary metabolites. Owing to their diverse characteristics, terpenes have been used in numerous applications because they perform specialized chemical functions to protect plants from abiotic and biotic stresses (Roba, 2020). The term “terpene” originates from the Latin word *terebinthina,* which denotes turpentine, a resinous material extracted from trees belonging to the genus, which are related to pine trees (Abdallah and Quax, 2017). Terpenes are an enormous class of naturally derived chemicals comprising over 30, 000 members and are common sources for a wide variety of purposes (Kennedy and Wightman, 2011). Although they occur in all organisms, they are mostly found in higher plants (Zwenger and Basu, 2008). According to a study by Wallach in 1887, these are simple hydrocarbons comprising isoprene units (C_5_H_8_), often referred to as the backbone of the terpenes. Two precursor molecules are responsible for terpene biosynthesis: the isopentenyl diphosphate (IPP) unit and its isomer, dimethylallyl diphosphate (DMAPP) (Abdallah and Quax, 2017). Two autonomous pathways are involved in the production of these two molecules. The mevalonate (MVA) and 2C-methyl-D-erythritol-4-phosphate (MEP) pathways. Both IPP and DMAPP undergo further rearrangement, repetition, and cyclization to yield different terpenes (Figure 1) (Tholl, 2015; Block et al., 2019). With further modifications, they are converted into terpenoids, also known as oxygenated derivatives of terpenes, by changing the type of functional groups attached to them (Martins et al., 2017).

Terpenes have a variety of medical applications, but their antiplasmodial activity stands out because they work similarly to the widely used antimalarial drug chloroquine. Despite their widespread use, there is currently little scientific data on the effects of terpenes on the cardiovascular system, which restricts their prospective application as cardioprotective and/or cardiotherapeutic drugs (Cox-Georgian et al., 2019). Certain terpenes are widely used in natural folk medicine. Terpenes constitute the largest group of constituents in essential oils. Approximately a quarter of the terpene fractions of essential oils are composed of monoterpenes and sesquiterpenes (Alves-Silva et al., 2016). Additionally, they bring helpful insects such as pollinators and dispersers, as well as natural enemies of pests (Ninkuu et al., 2021). Additionally, a number of studies have shown that certain terpenes may lessen the symptoms of inflammation by reducing the release of pro-inflammatory cytokines such as interleukin 1, tumor necrosis factor-alpha, and nuclear transcription factor-kappa B (Del Prado-Audelo et al., 2021). It has been extensively researched how plants produce terpenes to combat biotic (pathogenic bacteria, herbivore pests, and weeds) and abiotic (water, temperature, light, and salt) stressors (Ninkuu et al., 2021).

**FIGURE 2:**
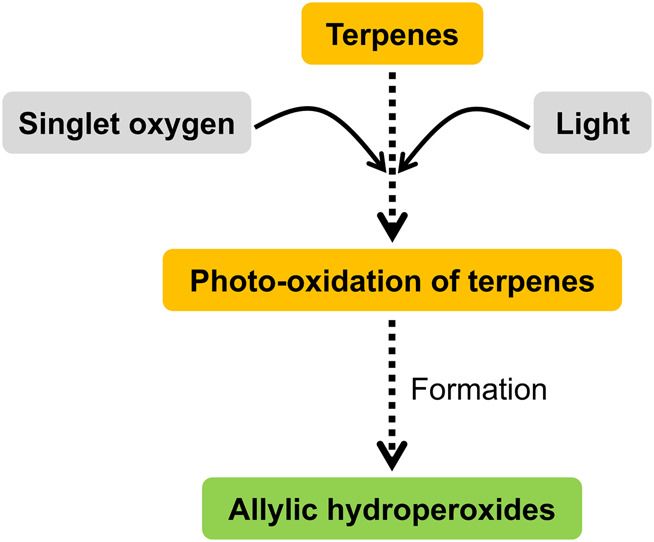
Oxygenation of terpenes.

The biochemistry of terpenes can be easily understood through their oxygenation, hydrogenation, and dehydrogenation to form terpenoids (Brahmkshatriya and Brahmkshatriya, 2013). Large amounts of terpenes are released into the troposphere by vegetation, where they react easily with ozone, OH, and NO_3_ radicals to produce a variety of oxidation products. Thus, the biochemical reaction pathways of terpenes include reactions of OH, O, and NO_3_ with simple alkenes, cycloalkenes, and conjugated dienes under tropospheric conditions. The oxidation products of terpenes are mainly carbonyl, carboxylic acids, alcohols, epoxides, esters, nitrates, and peroxynitrates (Figure 2), which are minor products (Calogirou et al., 1999). Terpene epoxides are thought to be promising primary intermediates in the synthesis of several green polymers, such as epoxy resins, polycarbonates, nonisocyanate polyurethanes, and even certain polyamides (Mahamat Ahmat et al., 2021). Terpenes were hydrogenated at temperatures as high as 200°C and 25 bar. In high yields and purities, the terpenes were converted to 2,6-dimethyloctane, farnesane, and squalane (Martinez-Botella et al., 2023). The purpose of hydrogenating terpenols is to eliminate double bonds. Direct hydrogenation using pressurized hydrogen is possible. To eliminate the double bonds in terpenols, a safer method is to use perhydrogenated cyclic hydrocarbons for transfer hydrogenation. In this process, virtual hydrogen is held inside the hydrogen donor molecule (Vostrikov et al., 2024). The presence of at least one double bond facilitates terpene dehydrogenation. The process of dehydrogenating terpenes and steroids to produce aromatic chemicals is reviewed, along with the current applications of some of the products. The underlying carbon structures of natural compounds, especially terpenoids and alkaloids, can be clarified using this technique before spectroscopic and crystallographic techniques take over (Yalın et al., 2023).

Terpenes are phytochemically important for plant defense against invasive plants and herbivores, disease resistance, chemical signaling and communication, defense against photooxidation, plant-environment mediation, thermoprotection, and pollinator attraction (Cox-Georgian et al., 2019). A type of resin that contains terpenes is secreted by capitate-stalked glandular hairs that are found on flower bracts, and to a lesser degree, by capitate sessile and bulbous trichomes that are also found in other vegetative organs. Additionally, yellowish and pungent essential oils have been extracted from the volatile terpenes of hemp glandular trichomes (Mazzara et al., 2022). Terpenes give plants their flavor, aroma, and pigmentation, which is why they are commercially used as food colors and fragrances. Natural terpenoids present new opportunities for the discovery of drugs with fewer side effects. Furthermore, terpenoids have been closely linked to a variety of biological activities, including anti-inflammatory, anti-allergic, antibacterial, antifungal, antioxidant, antiangiogenic, and antimetastatic properties. They usually comprise essential oils that are economically valuable in the form of scents and flavors. In the food sector, they are widely used as natural flavoring agents. They are utilized as natural food preservatives, natural flavorings, novel anticancer drugs, insecticides, and herbicides in agriculture and as raw materials for industrial chemical manufacturing (Parekh et al., 2024).

### 1.1 Terpenes in food industry

Terpene compounds can have a wide range of shapes and forms. Terpenes in fermented foods can serve both as culinary flavors and health benefits. Terpenes along with alcohols, acids, esters, amino acids, hydroxyl compounds, acetals, compounds containing nitrogen and sulfur, furan compounds, phenolic compounds, and ethers are among the more than 300 trace components of the primary flavoring molecules found in Chinese baijiu (Zheng et al., 2017). According to the literature currently available, terpene-derived essential oils have antibacterial and food-preservative qualities that make them a viable option for use in the food sector. A study by Fabio et al. (2024) explored the antimicrobial properties of combining essential oils and terpenes with supercritical carbon dioxide (sc-CO_2_) to preserve chicken breast meat (Fabio et al., 2024). Because of their hydrophobic structure, terpenoids are frequently lipophilic, allowing them to disrupt cell membranes and inhibit enzymes involved in energy metabolism and DNA synthesis, which can compromise cell viability. For example, α-pinene and limonene can suppress the activity of *E. coli* and *Staphylococcus aureus*. Conversely, some terpenoids function as antioxidants by neutralizing free radicals and safeguarding food from oxidative harm (Wang et al., 2025).

### 1.2 Terpenes in pharmaceutical industry

Like many other natural compounds, isoprenoids have biological activity that have been used to treat and prevent diseases in humans. Terpenes are also advertised as drugs that improve skin penetration, and are used to treat and prevent a number of inflammatory diseases. The cellular and molecular mechanisms underlying the pharmacological actions of hundreds of isoprenoid substances and their derivatives have been investigated. Forest bathing, which was later found to have positive health effects due to the aerosolic release of isoprenoid chemicals by forest trees, is where the concepts of aromatherapy and aerosol therapy originated (Tetali, 2019). Terpenes are mostly used as novel cancer therapies in the pharmaceutical industry. For instance, elemene, a naturally occurring anticancer substance with somewhat harmful side effects, prevents brain tumors, liver cancer, lung cancer, and nasopharyngeal carcinoma (Fan et al., 2023).

## 2 Types and classification of terpenes

**FIGURE 3:**
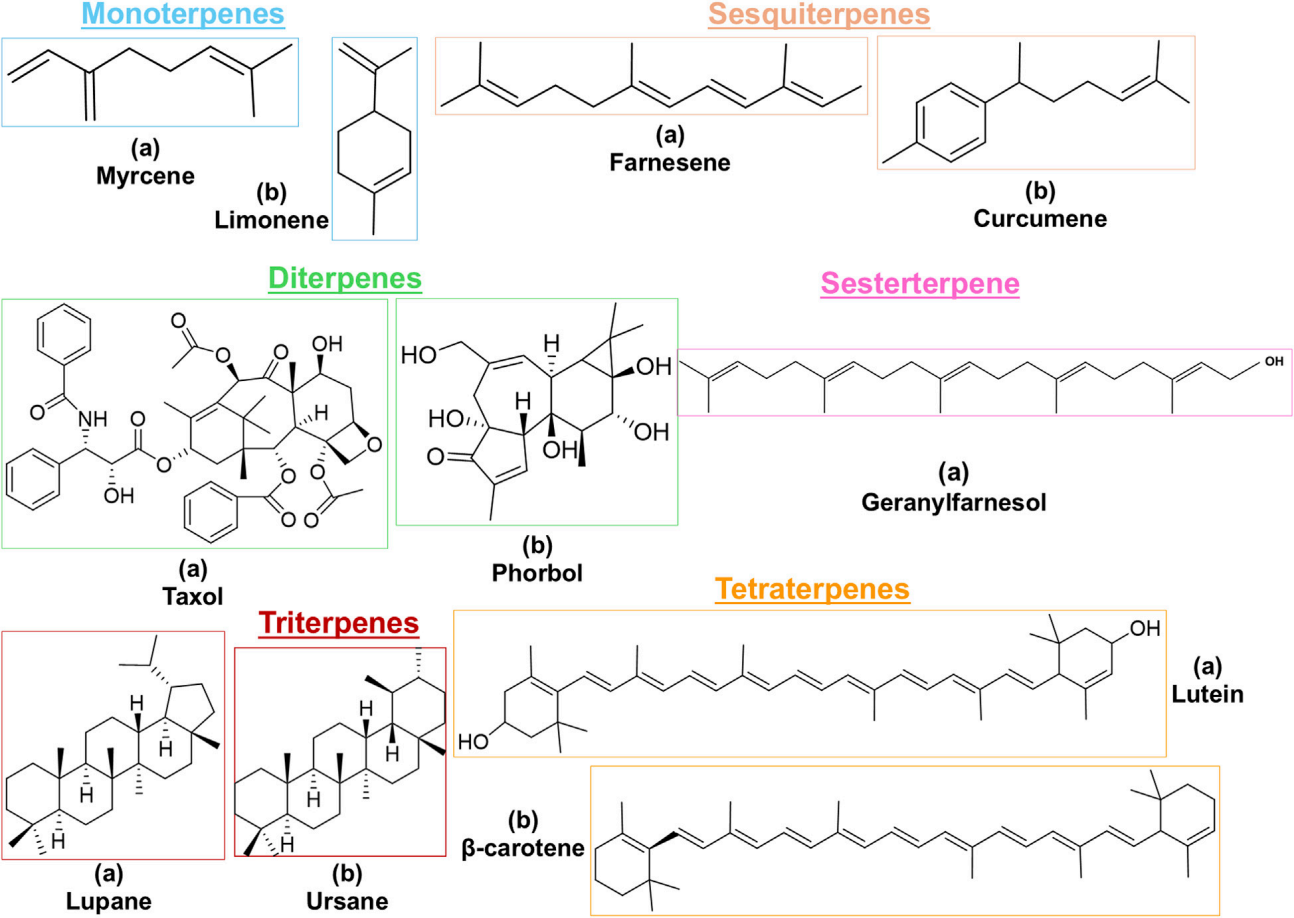
Based on classification of terpenes the structural form of the most common terpenes.

Terpenes can be primarily classified into monoterpenes (C_10_H_16_), sesquiterpenes (C_15_H_24_), diterpenes (C_20_H_32_), sesterpenes (C_25_H_40_), triterpenes (C_30_H_48_), tetraterpenes (C_40_H_64_), and polyterpenes (C_5_H_8_) n based on the number of isoprene units (Figure 3). The table below provides a brief description of the classification of terpenes (Mishra et al., 2024).

### 2.1 Monoterpenes (C_10_H_16_)

They are regarded as the smallest group of terpenes, with boiling points ranging from 150°C to 185°C, and consist of two isoprene units (Cox-Georgian et al., 2019). They are the primary components of many essential oils, fragrances, and flavors extracted from natural sources such as plants, fruits, vegetables, herbs, and spices (Hüsnü et al., 2007). Monoterpenes are synthesized by monoterpene synthases using geranyl pyrophosphate (GPP) as a substrate. Structurally, these compounds are further classified as acyclic, monocyclic, or bicyclic compounds synthesized by monoterpene cyclases (Loza-Tavera, 1999). These compounds are primarily responsible for attracting pollinators and protecting plants from predators. However, some studies have suggested their roles in the flowering process of plants (Sobral et al., 2014). Naturally occurring monoterpenes such as myrcene, thymol, limonene, linalool, and eugenol are the major phytoconstituents of many fruits, herbs, and oils. In addition to their distinct roles in plants, monoterpenes have promising biological properties, including cardioprotective, antimicrobial, anti-inflammatory, and antioxidant effects (Table 1) (Zielińska-Błajet and Feder-Kubis, 2020; Yang et al., 2023). Hinokitiol, a natural monoterpenoid extracted from the heartwood of *Calocedrus formosana*, has shown notable anticancer effects against a range of cancers, including those of the colon, cervix, breast, bone, endometrium, liver, prostate, mouth, and skin. Its anticancer mechanisms include triggering DNA damage, encouraging autophagy, and halting the cell cycle, which ultimately leads to apoptosis in cancer cells. Furthermore, hinokitiol has been found to prevent cancer cell migration and metastasis by inhibiting matrix metalloproteinases (MMPs) and influencing critical signaling pathways like ERK and AKT. These diverse actions make hinokitiol a promising candidate for developing new anticancer treatments (Bhuia et al., 2025).

**TABLE 1:** Monoterpenes and some commonly employed natural terpenes and derivatives and their sources and applications in medicine.

Monoterpenes				
Name of terpenes	Source	Medical applications	Chemical structure	References
Myrcene	*Cannabis sativa, Humulus lupulus, Houttuynia chordata, Cymbopogan citratus, Mangifera indica, Myrcia magnifolia, Verbena officinalis, Pimenta racemose* and *Elattaria cardamomum*	Osteoarthritis, anti-inflammatory, anti-catabolic, antifungal, antibacterial, and anti-nociceptive properties	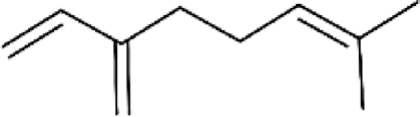	Surendran et al. (2021)
Limonene	Oils of *Citrus sinensis, Citrus paradisi, Citrus limon,* and *Anethum graveolens L. (dill)*	Anti-inflammatory, anti-oxidant, antinociceptive, anticancer, anti-diabetic, anti-hyper analgesic, anti-viral, and gastro-protective effects	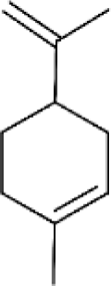	Mukhtar et al. (2018)
Linalool	*Mentha spicata, Coriandrum sativum, Ananas comosus, Ribes nigrum, Ocimum basilicum,* and *Ocimum tenuiflorum*	Anti-inflammatory, anticancer, anti-hyperlipidemic, antimicrobial, antinoceptive, analgesic, anxiolytic, anti-depressive and neuro-protective properties	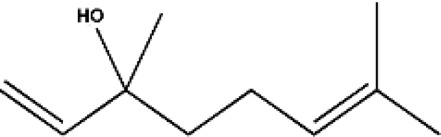	Pereira et al. (2018)
α-pinene	Oils of aromatic plants like *Eucalyptus* and *Salvia rosmarinus* oils	Antioxidant, anticancer, anticonvulsant, antiulcer, antihypertensive, anti-nociceptive, antibiotic resistance modulation, anticoagulant, antitumor, antimicrobial, antimalarial, antioxidant, anti-inflammatory, anti-Leishmania, and analgesic effects	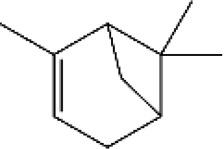	Salehi et al. (2019)
β-pinene	Essential oils of many plants and is majorly found in *Nepeta nepitella* and *Teucrium montanum*	H_2_O_2_-stimulated oxidative stress, pancreatitis, stress-stimulated hyperthermia, pulpal pain, breast cancer, antimicrobial, antimalarial, antioxidant, and leukemia	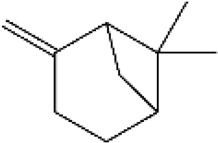	Salehi et al. (2019)
Eugenol	*Syzygium aromaticum buds, Ocimum tenuiflorum leaves, Curcuma longa, Origanum vulgare, Zingiber officinale,* and *Thymus vulgaris*	Commonly used for skin infections, skin lesions and inflammatory disorders, treatment of toothache and pulpitis, reproductive disorders, nervous system disorders, blood glucose and cholesterol irregularities, microbial infections, tumorigenesis, hypertension, inflammations, and digestive complications	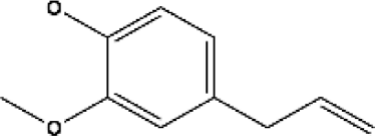	Nejad et al. (2017)
Camphor	Wood of *Cinnamomum camphora*	Possess properties like antitussive (anti-cough), anti-inflammatory, treatment of warts, cold sores, hemorrhoids, osteoarthritis, and allergies	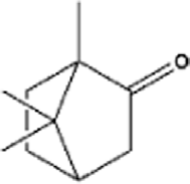	Lee et al. (2022)
Menthol	*Mentha arvensis* and many other mint plants	Antipruritic, antiseptic, analgesic, cooling effect, insomnia, irritable bowel syndrome, and anticancer	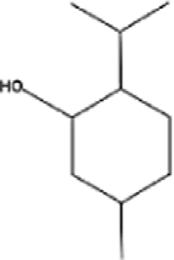	A Farco and Grundmann (2013)
Thymol	Essential oil of thyme (*Thymus vulgaris L.,* Lamiaceae)	Anti-inflammatory, antiviral, antibacterial, and anti-septic, treatment of burns and ulcerations	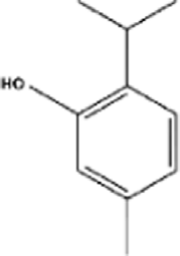	A Farco and Grundmann (2013)
Sabinene	*Picea abies, Piper nigrum,* and *Myristica fragrans*	Possesses anti-inflammatory, and antimicrobial properties, treatment of bladder disease, asthma, scabies, hemorrhoids, bronchitis, throat inflammation	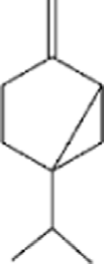	Sharma et al. (2019b)
Alpha phellandrene	*Eucalyptus phellandra* now known as *Eucalyptus radiata*	Anticancer (liver cancer cells), anti-oxidant, antibacterial insecticidal, anti-inflammatory, wound healing, analgesic, and neuronal responses	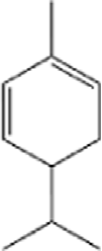	Thangaleela et al. (2022)
Geraniol	Essential oils of (*Monarda fistulosa, ninde, rose, palmarosa*	Anticancer (prostate, bowel, liver, kidney, and skin cancer), anti-oxidant, anti-inflammatory and antimicrobial activity	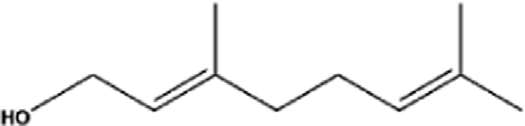	Thangaleela et al. (2022)
Camphene	*Chrysanthemum, Zingiber officinale, Rosmarinus offinialis*	Anti-inflammatory, antioxidant, antifungal, anticancer, antiparasitic, antidiabetic, and hypolipidemic activities	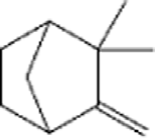	Mączka et al. (2020)
Ocimene	*Mentha, Petroselinum crispum, Ocimum basilicum, Artemisia dracunculus, kumquats, Lavandula, Humulus and Citrus bergamia*	Anti-oxidant, antibacterial, and anticancer effect against (oral, liver, lung, colon, melanoma, and leukemic cancer cells)	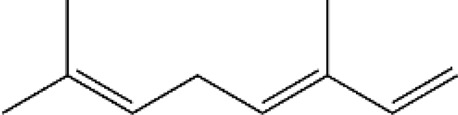	Lee et al. (2022)

### 2.2 Sesquiterpenes (C_15_H_24_)

This class of terpenes is a much larger group of compounds than monoterpenes and consists of three isoprene units (Hachlafi et al., 2023). Sesquiterpenes are considerably more constant than monoterpenes, originate from many plants, fungi, and insects, and are known to control activities such as defense mechanisms and metamorphosis (Tsang et al., 2020). Usually, these compounds are synthesized in the cytosol of higher plants *via* sesquiterpene synthase (STS) using isopentenyl pyrophosphate (IPP) and dimethylallyl pyrophosphate (DAMPP), and can be monocyclic, bicyclic, or tricyclic in nature (Koroch et al., 2007; Sallaud et al., 2009). Extracted from many natural sources, these compounds have an enormous variety of pharmacological potentials, including anti-inflammatory, antimalarial, and antitumor effects. Owing to their characteristic features, sesquiterpenes have been widely investigated for their potential use as natural drugs (Table 2) (Shoaib et al., 2017). The most common sesquiterpenes were caryophyllene, curcumene, and farnesene. β-Caryophyllene, a bicyclic sesquiterpene commonly found in a variety of plant essential oils, demonstrates notable biological activities, including anti-inflammatory, antioxidant, antimicrobial, and anticancer properties. Its ability to selectively activate the CB2 cannabinoid receptor allows it to modulate immune responses and reduce inflammation without causing psychoactive effects. Recent research has emphasized its neuroprotective and metabolic regulatory functions, highlighting its potential for therapeutic use (Iorio et al., 2022).

**TABLE 2:** Diterpenes, triterpenes, sterpenes, sesquiterpenes, and some commonly emlyoed natural terpenes and derivatives, as well as their sources and applications in medicine.

Name of terpenes	Source	Medical applications	Chemical structure	References
Diterpenes				
Totarol	Heartwood of *Podocarpus totara*	Anticancer (gastric cancer cells), antioxidant, anti-inflammatory, and antibacterial	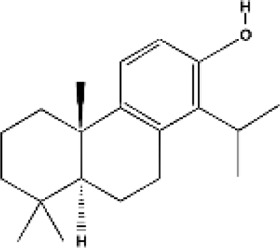	Jaiswal et al. (2007), Xu et al. (2019)
Taxol	Bark of *Taxus brevifolia*	Anticancer (breast, lung, and ovarian cancer) and antimicrobial properties	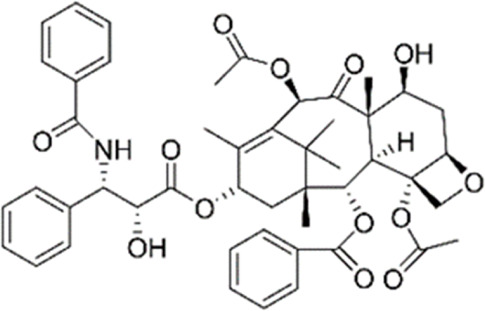	Young et al. (1992)
Abietanes	*Salvia, Tetradenia riparia* and *Euphorbia*	Anticancer antimalarial, anti-inflammatory, antioxidant, antiviral, antiulcer and antibacterial	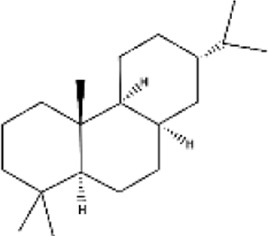	Gazim et al. (2014)
Carnosol	*Rosmarinus officinalis, alvia pachyphylla*	Anticancer, anti-inflammatory	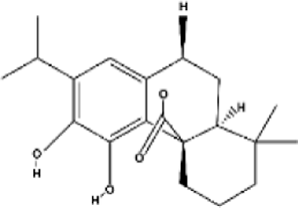	Johnson (2011)
Labdanes	Secondary metabolites in tissues of fungi, insects, marine organisms, and in essential oils, resins and tissues of higher plants	Anti-inflammatory, anticancer, antibacterial, antimalarial	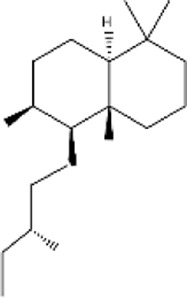	Chinou (2005), Matsingou et al. (2005), Villamizar et al. (2009)
Triterpenes				
Lupanes	Found in Fabaceae family	Anticancer, antibacterial, anti-inflammatory	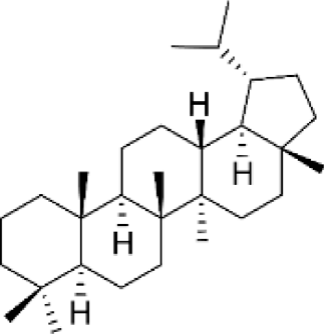	Tolstikova et al. (2006)
Ursanes	*Prunella vulgaris, Ocimum basilicum, agonia arabica and Aralia decaisneana*	Anticancer, antibacterial, anti-inflammatory	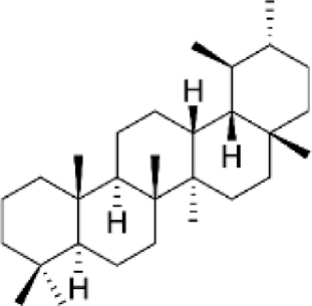	do Nascimento et al. (2014)
Glycyrrhizin	*Glycyrrhiza glabra*	Neurodegenerative disease, anti-inflammatory, antimalarial, antiviral, antioxidant	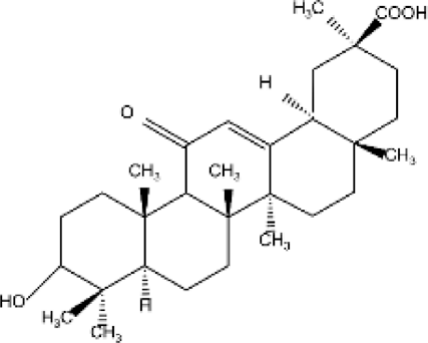	El-Saber Batiha et al. (2020)
Sesterterpenes				
Phylloquinone	Spinach, collards, kale, and broccoli, oybean, canola, cottonseed, and olive oils	Blood clotting, Bone and vascular metabolism	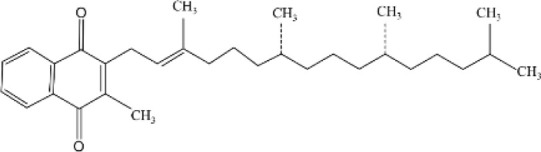	Basset et al. (2017)
Ophiobolins	Fungal pathoges *Helminthosporium orizae, Ophiobolus miyabeanus, Drechslera gigantea*	Anticancer, antifungal, nematocidal, anti-influenza	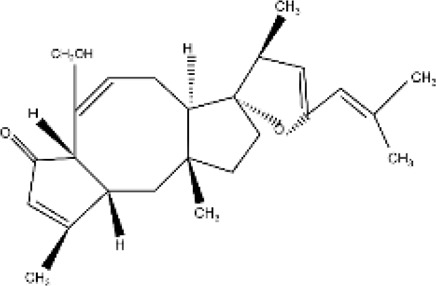	Tian et al. (2017)
Geranylfarnesol	Insect wax	Anticancer, antifungal, antibacterial	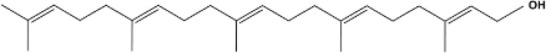	Bonikowski et al. (2015)
Sesquiterpenes				
β-caryophyllene	Majorly found in *Origanum vulgare, Cinnamon* spp.*, Piper nigrum,* and *Syzygium aromaticum bud essential* oil	Anticancer, cardio-protective, hepato-protective, gastro-protective, neuro-protective, nephron-protective, anti-inflammatory and immune-modulatory actions	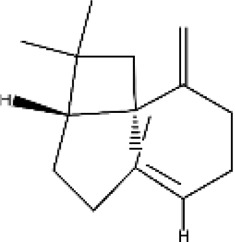	Dahham et al. (2015), Francomano et al. (2019)
α- Humulene	*Humulus lupulus*	Active as an anti-inflammatory, anticancer, antimicrobial agent, and arthritis	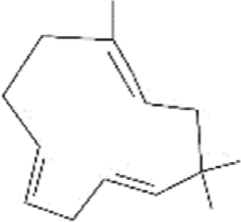	Rogerio et al. (2009)
Nerolidol	*Rosa rubiginosa, Cymbopogon nardus, Jasminum, Zingiber officinale, Melaleuca alternifolia*	Antimicrobial, antibiofilm, anti-oxidant, anti-parasitic, skin-penetration enhancer, skin-repellent, anti-nociceptive, anti-inflammatory and anticancer	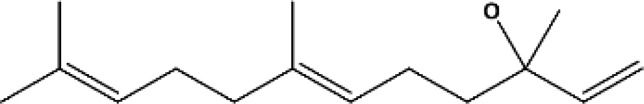	Chan et al. (2016)

### 2.3 Diterpenes (C_20_H_32_)

Diterpenes, which are composed of four isoprene units, are the most abundant secondary metabolites in plants and fungi (Sobral et al., 2014). The biosynthesis of diterpenes involves the formation of the precursor geranylgeranyl diphosphate (GGPP), catalyzed by GGPP synthase, followed by the formation of a carbon backbone, which is catalyzed by diterpene synthases (DTSs), and further structural modifications catalyzed by cytochrome P_450_ oxygenases (CPYs or P450s) (Ashour et al., n. d.; Bathe and Tissier, 2019). Modifications in the side chains of diterpenes have been reported to be catalyzed by prenyltransferases (PTs) as well as DTSs (Bathe and Tissier, 2019). These compounds are responsible for many events in plants, such as regulating germination, flowering, and the switching of reproductive cycles from sexual to asexual reproduction. In addition to their diverse properties in plants, diterpenes are a group of bioactive chemicals with prominent features such as cytotoxicity, antimicrobial, anti-inflammatory, and antitumor effects (Table 2). The most commonly used diterpenes are taxol, phorbol, cembrene, and Taxusin. Coronarin D, a diterpene of the labdane type, shows significant anticancer potential by employing various mechanisms such as triggering apoptosis, halting the cell cycle, and preventing metastasis. Its diverse actions underscore its promise as a leading candidate for creating new anticancer drugs. Coronarin D demonstrates anticancer properties by triggering apoptosis *via* mitochondrial pathways, leading to G2/M cell cycle arrest and enhancing ROS production. Additionally, it suppresses NF-κB signaling, reduces MMPs expression, and influences EMT markers, collectively hindering tumor growth and metastasis (Bailly, 2020). Diterpenoids sourced from the Lamiaceae family have shown potential as effective multitarget agents for combating leishmaniasis and Chagas disease. In particular, abietane-type diterpenoids, including royleanones and derivatives of dehydroabietic acid, have proven to be highly effective against both Leishmania and Trypanosoma cruzi, impacting various life stages of these parasites. These compounds work by disrupting mitochondrial function, triggering apoptosis, and influencing oxidative stress pathways, thereby offering a comprehensive approach to treating these neglected tropical diseases (de Menezes et al., 2025).

### 2.4 Sesterterpenes (C_25_H_40_)

Sesterterpenes are a ubiquitous group of secondary metabolites comprising twenty-five isoprene units, and are primarily found in plants, insects, fungi, and marine organisms (Negi et al., n. d.). Sesterterpenes are biosynthesized from two common carbon precursors, IPP and its isomer DMAPP, similar to other known classes of terpenes. The cyclization of terpene synthases (TPs) with further alterations and modifications, such as acylation, glycosylation, and oxidation, leads to the formation of sesterterpenes. These compounds can be further divided into linear and carbocyclic types (Chen Q. et al., 2021). To date, approximately 1,000 sesteterrpenes have been isolated from different natural sources (Table 2) (Shirley et al., 2018). Geranylfarnesol, ophiobolins and manoalideare the most common naturally occurring sesterterpenes. Recent studies have highlighted the therapeutic promise of rare sesterterpenoids extracted from the soil-derived fungus Aspergillus variecolor strain SDG. Notably, stellatic acid showed strong antioxidant properties and exhibited significant cytotoxicity against a range of cancer cell lines, such as HeLa, HepG2, MCF7, and A549, with IC_50_. Furthermore, stellatic acid demonstrated a tenfold greater inhibitory effect on yeast α-glucosidase compared to mammalian α-glucosidase. Molecular docking studies also indicated a stronger binding affinity of stellatic acid to yeast α-glucosidase, suggesting its potential as a lead compound for the development of new antioxidant, anticancer, and antidiabetic treatments (Kumar et al., 2025).

### 2.5 Triterpenes (C_30_H_48_)

Triterpenes are among the largest, most varied, and most complex classes of terpenes with six isoprene units, and are derived from the linear isoprenoid substrate 2,3-oxidosqualene (Hussain et al., 2012). These compounds exhibit distinct properties because they are rich in methyl groups and can be oxidized into several compounds such as aldehydes, alcohols, and carboxylic acids (Parveen, n. d.). Synthesized *via* the mevalonic acid (MVA) pathway, approximately 20,000 types of triterpenes have been reported to date, more than 100 of which are currently derived from a vast majority of plant sources. Oxidosqualene cyclases (OSC) are enzymes responsible for the cyclization of 2,3-oxidosqualene-generating sterols or triterpenes, which serve as biological precursors for triterpene synthesis (Thimmappa et al., 2014). Most triterpenes have been reported to possess numerous biological properties, including anticancer, antiviral, antibacterial, and anti-inflammatory activities. Lupane, ursane, oleanane, and cucurbitane are triterpenes with numerous applications (Table 2) (Nazaruk and Borzym-Kluczyk, 2015). Ursolic acid triterpene derivatives possess a broad spectrum of pharmacological effects, including anti-inflammatory, anticancer, antimicrobial, and antioxidant properties. These activities are largely facilitated by mechanisms such as the inhibition of NF-κB and MAPK signaling pathways, the induction of apoptosis through mitochondrial routes, and the reduction of oxidative stress and pro-inflammatory cytokines. Modifying the structure of ursolic acid improves its bioavailability and effectiveness, making these derivatives attractive candidates for therapeutic development. Additional research is needed to refine their pharmacokinetic characteristics and confirm their effectiveness in clinical environments (Khwaza and Aderibigbe, 2024).

### 2.6 Tetraterpenes (C_40_H_64_)

Generally known as ‘carotenoids,’ these compounds consist of eight isoprene units. In plants, algae, and cyanobacteria, tetraterpenes mainly contribute to the protection of plants against free radicals, antioxidative activities, and often light trapping (Ninkuu et al., 2021). These compounds belong to the most studied group of natural compounds with over 750 members. Many of these compounds are highly unsaturated and are extremely difficult to isolate and purify (Maoka, 2020). β-Carotene is one of the most prominent and crucial compounds among all tetrapenes because it acts as a precursor for the production of vitamin A in mammals. Other important tetrapenes include lutein, lycopene, and α-carotene. A recent double-blind, randomized controlled trial explored the impact of lutein supplementation on the metabolic health of adults with central obesity. Over a period of 32 weeks, participants were given either 10 mg of lutein daily or a placebo. Those in the lutein group showed notable decreases in plasma total cholesterol, low-density lipoprotein cholesterol (LDL-C), apolipoprotein B (ApoB), and malondialdehyde levels compared to the placebo group. Furthermore, there was a significant reduction in advanced glycation end products (AGEs), such as carboxyethyl lysine (CEL), carboxymethyl lysine (CML), and methylglyoxal hydroimidazolone (MG-H1). An increase in plasma lutein concentration was positively associated with improvements in the skin carotenoid index and inversely related to AGEs levels. These results indicate that regular lutein consumption may improve metabolic health by lowering oxidative stress and lipid levels in individuals with central obesity (Zhou et al., 2025).

## 3 Terpenes in metabolic disorder

The association between drug discovery and plants has been well documented since humankind came into existence (Parveen et al., 2015). During the last 20 years, lethal side effects of synthetic drugs have attracted considerable interest from researchers in plant-based formulations worldwide. A combination of coexisting metabolic disorders, known as metabolic syndrome, increases the risk of type 2 diabetes, heart disease, and stroke. Plant-derived secondary metabolites may serve as effective agents for the management or avoidance of metabolic syndrome with various modes of action that may potentiate each other’s activity or have a synergistic effect, providing greater benefits than a single chemical entity (Graf et al., 2010). Terpenes have been shown to affect metabolic status.

### 3.1 Terpenes in type 2 diabetes

The hallmark of type II diabetes is hyperglycemia, which results from impaired insulin signaling. Triterpenes may be used as therapeutic agents for diabetic retinopathy, peripheral neuropathy, and kidney failure by blocking many pathways associated with hyperglycemia and its consequences, according to another study (Roy et al., 2024). The compact molecular structure, limited ramification, and reduced symmetry of sesquiterpenoids make them more effective for treating diabetes. In a different study, oral dlimonene treatment decreased plasma glucose and glycosylated hemoglobin levels and the activities of gluconeogenic enzymes such as fructose 1, 6 bisphosphatase, and glucose 6-phosphatase, while increasing the activity of the glycolytic enzyme, glucokinase, and liver glycogen in diabetic rats (Panigrahy et al., 2021). Twelve terpenoids were tested in mice with normoglycemia and streptozotocin-induced diabetes to treat type 2 diabetes mellitus. Among these, in streptozotocin-induced diabetic mice, geranic acid, citral citronellic acid, and three sesquiterpenes-farnesal, farnesol, and farnesyl acetate exhibited antihyperglycemic action (Valdes et al., 2019).

### 3.2 Terpenes in obesity

Obesity and overweight are major global health and financial issues. Every body system may be affected by this medical condition, which can result in problems such as cancer, diabetes, metabolic syndrome, dyslipidemia, cardiovascular disease, and hypertension. Potential sources of anti-obesity compounds include diterpenes and their derivatives (Islam et al., 2021). According to a recent study by Huang et al., in fatty liver cells generated by oleic acid, the diterpene lactone ginkgolide C, which is derived from Ginkgo biloba, decreases lipid accumulation and promotes lipolysis *via* the sirt1/AMPK pathway (Lee et al., 2018). According to a recent study, the pharmacological activity of terpene-containing analogs of glitazarsin in obese and type 2 diabetic mice showed that administering a dose of 30 mg/kg for a month increased their catabolism, which decreased the amount of triglycerides in the liver and adipose tissue. This was followed by a hypoglycemic effect that improved insulin sensitivity in mice (Blokhin et al., 2023).

### 3.3 Terpenes in fatty liver diseases

Infectious diseases have gradually become the focus of metabolic disease research in the epidemiology of liver diseases. Numerous bioactive terpenoids found in herbal plants can modify metabolic conditions specific to the liver, including chronic liver disease, nonalcoholic fatty liver, nonalcoholic steatohepatitis, and even liver damage caused by drugs, alcohol, and hyperglycemia. The mechanism of insulin resistance may be partially responsible for the favorable correlation between serum terpenes and metabolic syndrome (Wang et al., 2021). *Melaleuca alternifolia, Salvia officinalis*, and *Carthamus tinctorius* all contain α-terpineol, a monoterpene alcoholic that is frequently used as an essential oil and flavoring in food. Terpenes significantly increased HDL-C levels and significantly lowered ALT, AST, and ALP enzymatic activity, as well as total serum bilirubin levels in hyperlipidemic mice (Choi et al., 2013). According to a number of studies, liver toxicity, which is primarily caused by the production of reactive metabolites, elevated levels of reactive oxygen species, and compromised antioxidant defense, was demonstrated by certain monoterpenes (such as pulegone, menthofuran, camphor, and limonene) and sesquiterpenes (such as zederone, germacrone) (Zárybnický et al., 2018).

## 4 Pharmacological activity of terpenes

Terpenes have been shown to exhibit antibacterial properties against bacteria that are susceptible and resistant to antibiotics, mostly through their capacity to enhance cell rupture and impede the synthesis of proteins and DNA (Masyita et al., 2022). Terpenoids have hypoglycemic properties, enhance transdermal absorption, prevent and treat cardiovascular disorders, and have anticancer, anti-inflammatory, antibacterial, antiviral, and antimalarial properties. Terpenoids also have numerous potential uses including insect resistance, immunoregulation, antioxidation, antiaging, and neuroprotection (Yang et al., 2020).

### 4.1 Terpenes as antibacterial agents

#### 4.1.1 Action against antimicrobial resistance (AMR)

Antimicrobial resistance (AMR) is becoming a worldwide risk owing to both extrinsic and intrinsic factors, and the transfer of resistance genes is often regarded as the major cause of AMR. Microbial resistance not only leads to the development of new resistant strains but also contributes to the poor efficacy of antibiotics, thereby drastically reducing their therapeutic effects. To overcome this obstacle, the discovery of plant-derived molecules has shown a commendable effect against AMR strains. Talking about plant secondary metabolites, terpenes are a group of highly classified molecules having an enormous range of biological properties. Numerous terpenes, including mono-, di-, and tri-terpenes, have been identified to alleviate this problem and to create reservoirs for new alternatives. Although the antibacterial mode of action of terpenes is mostly unidentified, some studies have suggested that terpenes and their derivatives can inhibit two of the most crucial steps important for bacterial survival: uptake of oxygen and oxidative phosphorylation, which directly affect the rate of respiration (Shaw and Ingraham, 1967). Structurally, terpenes exhibit lipophilicity/hydrophobicity, which results from their hydroxyl groups present in them. This property of terpenes often determines their antibacterial properties (Table 1) (Chen et al., 2016). Carvacrol, thymol, menthol, and geraniol, which belong to the monoterpene class, were found to be active against Gram-negative and Gram-positive bacterial strains. Geraniol increases the susceptibility of Gram-negative and multidrug-resistant strains of *Enterobacter aerogenes* by acting as an efflux pump inhibitor (Table 1) (Lorenzi et al., 2009). In addition, linalyl acetate, (+) menthol, and thymol were active against *Staphylococcus aureus* and *Escherichia coli*. Other monoterpenes, such as trans-cinnamaldehyde and (+)-carvone, have been reported to possess strong antibacterial activities against *S. typhimurium* and *E. coli* (Chouhan et al., 2017). Farnesol, an acyclic sesquiterpene, has been shown to possess moderate activity against *Streptococcus mutans*, *Streptococcus aureus* (Kossakowska-Zwierucho et al., 2020), and *S. epidermidis,* and has potential antibiofilm activity against *Streptococcus sobrinus* (Gomes et al., 2009). Potency as a single molecule as well as in combination therapy has been achieved by many terpenes, such as farnesol and xylitol, for treating atopic dermatitis (Mahizan et al., 2019). In addition to xylitol, farnesol in combination with amoxicillin, doxycycline, ceftazidime, and sulfamethoxazole-trimethoprim showed potent activity against *Burkholderia pseudomallei* (Castelo-Branco et al., 2016). In addition to sesquiterpenes, diterpenes have been reported to improve the antibiotic activity of methicillin-resistant *Staphylococcus aureus* (MR*SA*) and have been employed in combination therapies. For example, salvipisone and aethiopinone, isolated from the roots of Salvia sclerosa, show antibacterial and antibiofilm activities against *Staphylococcus aureus*, *Enterococcus faecalis,* and *Staphylococcus epidermidis*. In combination with antimicrobial drugs such as oxacillin, vancomycin, and linezolid, these two diterpenes exhibit potent effects against MR*SA* and methicillin-resistant *S. epidermidis* (MR*SE*) (Mahizan et al., 2019). Built from six isoprene units, oleanic acid, bonianic acid A, and B are triterpenes that show potent activity against *Mycobacterium tuberculosis*. In addition, it showed promising effects against MDR strains when combined with rifampicin, isoniazid, and ethambutol (Barbieri et al., 2017).

Cell membranes and vesicles are powerful tools that contribute to the pathogenicity of bacteria. The cell membrane is responsible for upholding the integrity of the cell and controlling the normal physiological functions of bacteria. Therefore, targeting the bacterial cell membrane often simultaneously promotes cell lysis and destroys bacterial cells. The lipophilic nature of terpenes is a major factor in bacterial cell destruction. Terpene derivatives have the potential to diffuse and pass through the phospholipid bilayer of bacterial cells and are considered a virtuous source of natural antibacterial agents (Aytar Çelik et al., 2023). Studies have reported the action of 1,8-cineole, a monoterpene extracted from *Eucalyptus globulus Labill*, which exhibits strong antibacterial effects against *Acinetobacter baumannii*, *Candida albicans*, methicillin-resistant *Staphylococcus aureus*, and *E. coli* by destroying their cell membranes. Other studies conducted by different researchers proved that when carvacrol, cinnamaldehyde, thymol, eugenol, and limonene were exposed against *Salmonella typhimurium*, *E. coli*, *Brochotrix thermophacta* and *Staphylococcus aureus* cells, commendable membrane damage was observed *via* scanning electron microscopy (SEM). Therefore, terpenes can be considered reliable agents that promote cell membrane damage and exert bacteriostatic effects (Khan et al., 2020; Mączka et al., 2020).

#### 4.1.2 Anti-quorum sensing activity

Quorum sensing (QS) is a mechanism through which intercellular communication occurs between bacterial cells. QS fuels the pathogenicity of bacterial cells and promotes bacterial resistance by passing through various signaling molecules. Achieving antibacterial effects by inhibiting quorum-sensing mechanisms is a highly effective step in the treatment of resistant bacterial strains (Sharma et al., 2020). Studies have shown that, at low concentrations, carvacrol and thymol can inhibit acyl homoserine lactone (AHL), a molecule produced by AHL synthase for the smooth construction of biofilms *via* a quorum-sensing mechanism (Deryabin et al., 2019). Cinnamaldehyde has also been reported to inhibit AHL and many other auto-inducers complicated by quorum sensing (Escobar-Muciño et al., 2022).

#### 4.1.3 Inhibition of ATP and its enzyme

ATP is one of the most prominent and direct sources of energy for all organisms and is necessary for the basic functionalization of cells. Terpene derivatives are highly active against cell membranes, resulting in a changeable concentration gradient inside and outside the cell, which eventually leads to disruption of the cell membrane (Dunn and Grider, 2020). Some terpene derivatives, such as eugenol and thymol, which target bacterial cell membranes, also show fungicidal activity against *C. albicans via* inhibition of H^+^-ATPase, leading to the acidification of intercellular components and simultaneous cell death (Huang et al., 2022). Reports have revealed that when *E. coli was* treated with the MIC of carvacrol and extracellular ATP concentrations were observed using a luminometer, it potently disrupted the cell membrane along with the release of ATP and potassium ions. The absorbance was measured at 260 nm (Huang et al., 2022).

#### 4.1.4 Action *via* inhibition of protein synthesis

Protein synthesis shares an inseparable bond with the basic physiological activities of bacteria. The antibacterial effects of terpenes can be achieved by inhibiting protein synthesis pathways. Some *in vitro* studies have demonstrated that cinnamaldehyde can reduce the assembly and binding of the filamenting temperature-sensitive mutant Z-type protein (FtsZ), a prokaryotic tubulin homolog responsible for regulating cell division. Moreover, cinnamaldehyde inhibits GTP hydrolysis, binds to FtsZ, and interferes with z-loop formation during cell dynamics (Huang et al., 2022). Recent studies based on *in vivo* assays have shown that cinnamaldehyde is an effective inhibitor of *S. typhimurium* (stFtsZ), with an inhibition rate of GTPase activity and polymerization of up to 70% (Yin et al., 2022).

#### 4.1.5 Efflux pump inhibitor

**FIGURE 4:**
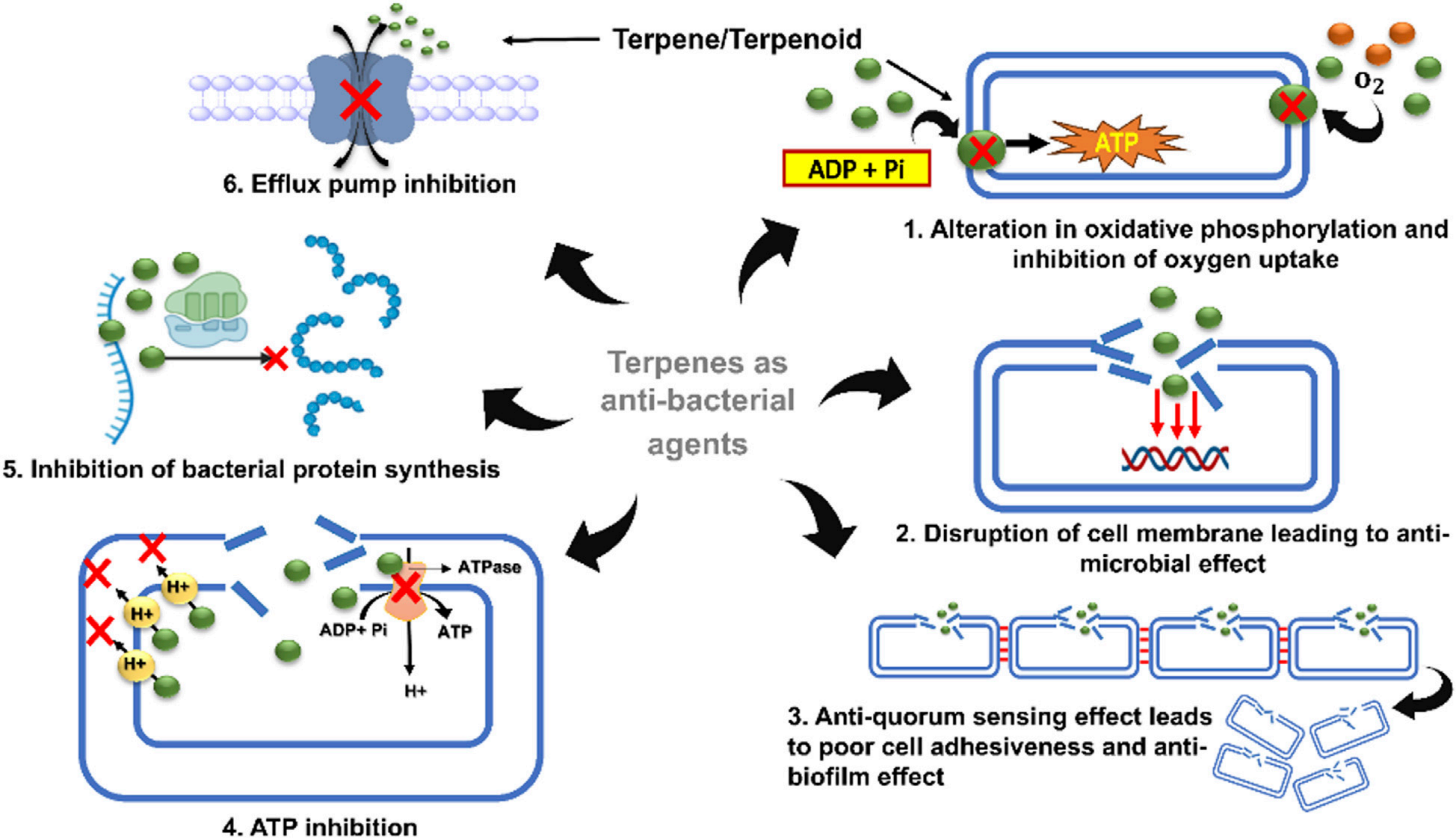
Representation of efflux pump inhibition by terpenes on the structural composition of the bacterial membrane by interaction with polysaccharides, phospholipids, fatty acids, and proteins.

The tremendous increase in antibiotic resistance has become a crucial challenge in modern pharmacotherapy. Molecular studies have provided significant evidence that the expression of bacterial efflux pumps is the underlying mechanism of multidrug resistance. Efflux pumps (EPs) that generate resistance often exhibit downregulated antibiotic effectiveness because they are unable to reach their target sites at inhibitory concentrations (Figure 4) (Muniz et al., 2021).

Frequent studies on the design of efflux pump inhibitors have demonstrated that NorA, TetK, and MepA are the major efflux proteins that contribute to bacterial resistance. In the search for natural efflux pump inhibitors, researchers have found that terpenes, flavonoids, tannins, and alkaloids exhibit impressive activities (Sharma A. et al., 2019). Among these, monoterpenes have been found to have a notable effect on the structural composition of bacterial membranes by interacting with polysaccharides, phospholipids, fatty acids, and proteins (Sikkema et al., 1994). According to various studies, carvacrol, thymol, and estragole are the most frequently studied terpenes, and *S. aureus* is the most frequently examined species of bacteria with respect to their action against efflux pumps (Gunes et al., 2016). It has been reported that both carvacrol and its lipid nanocapsules interfere with the efflux mechanisms of *A. baumannii.* In addition, carvacrol inhibits the overexpression of TetK, NorA, and MsrA proteins responsible for efflux mechanisms in *S. aureus* (Montagu et al., 2016). Many other studies have demonstrated a detailed mechanism by which terpenes can inhibit the NorA-mediated efflux system and can be designed as effective antibacterial agents against multidrug-resistant bacterial strains (Gupta et al., 2016).

### 4.2 Terpenes as anticancer agents

#### 4.2.1 Induction of apoptosis *via* cell cycle arrest and inhibition of signaling pathways

**FIGURE 5:**
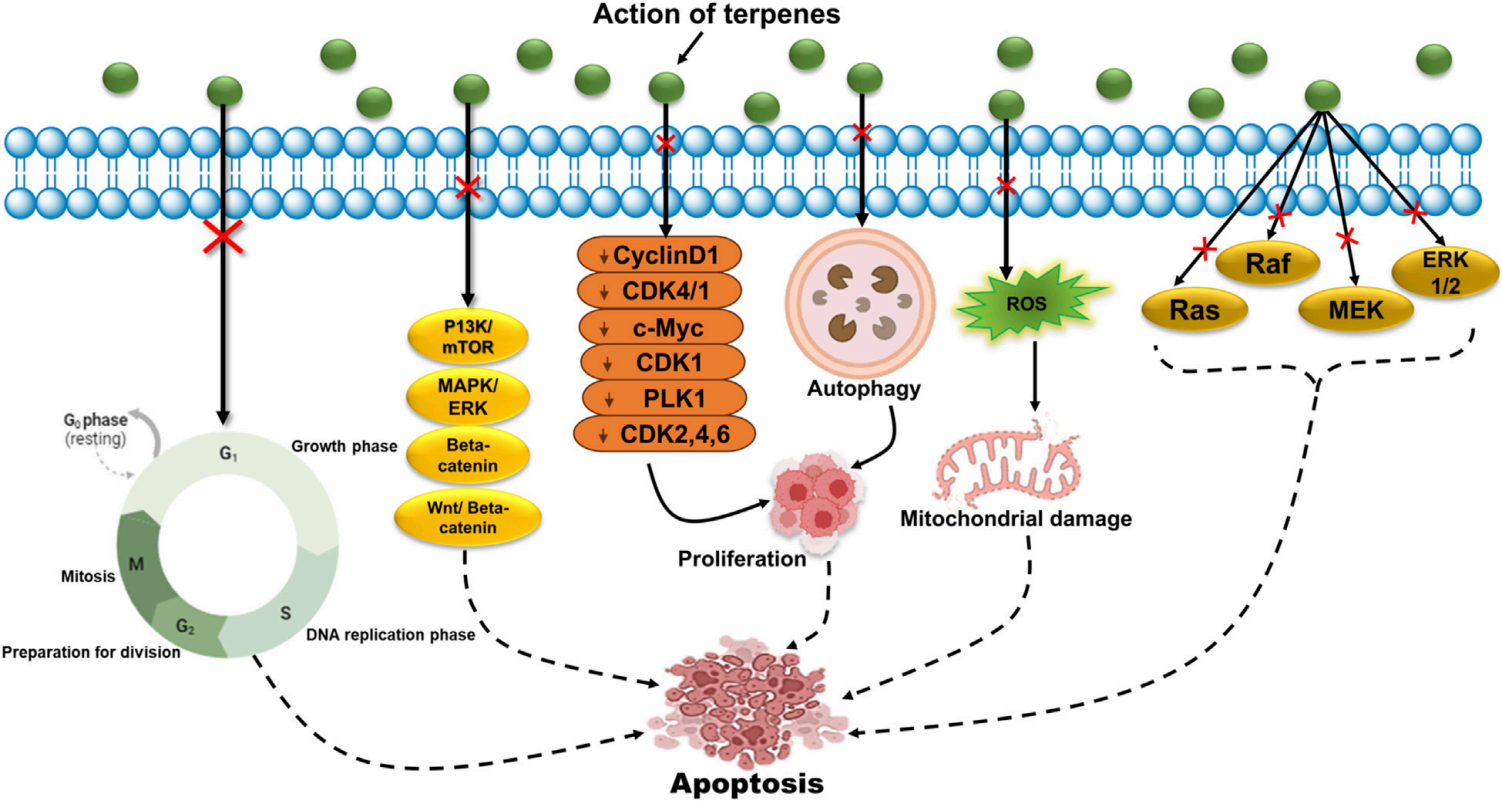
Diagram illustrating the role of terpenes in the epigenetic regulation of cancer cell inhibition.

In addition to their abundant activity against pathogenic microbes, terpenes and their derivatives have been demonstrated to be potential anticancer agents (Table 1). Evidence of terpenes as anticancer agents dates back to an early 1997 study, which concluded that a combination of mono-, di-, and sesquiterpenes has the potential to treat cancers of the colon, bones, prostate gland, and brain (Wróblewska-Łuczka et al., 2023). Among many other pathways, terpenes, such as carvacrol and thymol, can inhibit one of the most crucial signaling pathways responsible for cell proliferation and survival, the P13K/mTOR pathway, leading to the induction of apoptosis and cell death (Figure 5) (Xing et al., 2019; Sampaio et al., 2021).

It is also known to inhibit the MAPK/ERK pathway and induce cell cycle arrest at the G_2_/M phase, which is responsible for cell differentiation, proliferation, and survival (Yao et al., 2019). Many terpenes target regulatory proteins to inhibit cell cycle progression, such as taxol, which blocks cell division by stabilizing the microtubules (Stacchiotti and Corsetti, 2020). Carvacrol has been reported to be involved in cancer cell cycle arrest at the G_0_/G_1_ phase *via* downregulation of cyclinD1 and CDK4/1, whereas artesunate demonstrated antitumor effects by inhibiting β-catenin accumulation, which sequentially downregulates genes such as c-Myc and CDK1 (Zheng and Pan, 2018). It has also been found that menthol arrests the cell cycle at the G_0_/G_1_ phase in androgen-independent prostate cancer cells and the G_2_/M phase in PC3 prostate cancer cells by downregulating CDK2, 4, and six and downregulating downstream signaling of polo-like kinase 1 (PLK1), respectively (Figure 4) (Zhang et al., 2019; Kamran et al., 2022). The Wnt (wingless signaling)/β-catenin pathway is another major signaling pathway that contributes to tumor formation and the survival of cancer cells. Lupeol is a triterpene that possesses antitumor activity *via* inhibition of the Wnt/β-catenin pathway (Yu et al., 2021). Numerous studies have targeted terpenes that can prevent the expression of anti-apoptotic proteins, such as Bcl-xL, and simultaneously upregulate pro-apoptotic proteins, such as caspases and Bax, leading to apoptosis and cell death, such as betulinic acid, which can upregulate the intrinsic apoptotic pathway by downregulating anti-apoptotic proteins (Liu et al., 2019; Lee J.-Y. et al., 2021; Siraj et al., 2021; Kłos and Chlubek, 2022). Additionally, thymoquinone exerts efficacious effects against melanoma cells by suppressing Akt phosphorylation, which in turn increases the Bax/Bcl-2 ratio and potentially activates an intrinsic pathway for apoptosis, thereby inhibiting cell growth. These mechanisms are examples of terpenes possessing anticancer activities that induce apoptosis. However, many other pathways have been extensively studied to target cell cycle arrest and induce apoptosis using natural compounds to develop drugs with low toxicity and high efficacy (Wróblewska-Łuczka et al., 2023).

#### 4.2.2 Inhibition of cell proliferation

Terpenes and their derivatives exhibit anticancer activities by targeting various stages of cancer, of which cell proliferation is a major mechanism that might lead to the robust growth of cancer cells. Parthenolide is a sesquiterpene known to induce apoptosis and autophagy, leading to inhibition of cell proliferation by inhibiting the P13K/AKT/mTOR pathway in human hepatocellular carcinoma and breast cancer cells (Figure 5) (Kamran et al., 2022; Sahoo et al., 2025). Oleanolic acid exerts antitumor effects by initiating cell proliferation *via* autophagy-dependent mechanisms (Lee J.-H. et al., 2021). Many terpenes, such as MAPK/ERK, have also been reported to block some of the crucial signaling pathways responsible for cell proliferation (Sevastre et al., 2022). Additionally, various terpenes such as β-elemene, perillyl alcohol, and limonene inhibit the proliferation of melanoma cells by inducing apoptosis (Wróblewska-Łuczka et al., 2023). Furthermore, auraptene is a known terpene that inhibits cell proliferation by triggering the mTOR signaling pathway. According to an *in vivo* study, D-limonene is recommended as an anticancer agent because it effectively inhibits cell proliferation in mice with lymphoma by increasing nitric oxide (NO) production (Tayarani-Najaran et al., 2021; Kamran et al., 2022). Moreover, thymoquinone has been proven to be a potential anticancer agent because it inhibits many cancer progression pathways and signaling molecules, such as activation of the signal transducer and activator of transcription 3 (STAT3) signaling pathway, which contributes to cell proliferation and tumor survival (Asaduzzaman Khan et al., 2017; Farooqi et al., 2022). Betulinic acid was found to be effective against HeLa cell proliferation, followed by morphological changes induced by the upregulation of caspase 3 and initiation of apoptosis (Xu et al., 2017). Many other natural terpenes are yet to be explored for their anticancer effects to be designed as potent anticancer drugs.

#### 4.2.3 Modulation of gene expression

Epigenetic modifications often influence gene expression and regulation. Genetic changes such as chromosomal translocation, point mutations, deletion, amplification, and insertion activation can lead to uncontrolled cellular growth and invasiveness. Mutations in the gene p53 are the leading cause of unusual protein formation, and often play a key role in major disturbances in molecular processes related to p53 (Coyle et al., 2017; Ramazi et al., 2023). D-limonene inhibits HL-60 leukemia cell proliferation by altering p53 gene expression and downregulating Bcl-2 protein expression, simultaneously causing cell death (Guo et al., 2006; Chen Y. et al., 2021). Moreover, D-limonene treatment of skin tumors inhibited the expression of Ras, Raf, MEK, and ERK1/2 (Kamran et al., 2022). In contrast, menthol induces mitochondrial membrane depolarization in bladder cancer cells by increasing Ca^2+^ levels *via* overexpression of TRPM8, which leads to cancer cell death (Li et al., 2009; Ochoa et al., 2023). According to a study, auraptene, a monoterpene, was found to inhibit β-catenin T-cell factor (TCF) function while suppressing the overexpression of c-Myc proto-oncogene in human colorectal cancer cells (Rennoll and Yochum, 2015). Additionally, p21 overexpression and CDK1 and CDK4 downregulation were altered by perillic acid, which showed anticancer activity against colon cancer cells (Gunes-Bayir et al., 2022; Kamran et al., 2022). Similarly, the anticancer activity of carvacrol was investigated by evaluating apoptosis *via* modulation of p53, Bcl-2, and Bax protein expression (Figure 5) (Gunes-Bayir et al., 2022). Furthermore, investigations of triptolide as an anticancer drug have shown that it can induce apoptosis by inhibiting Akt, mTOR, and P70S6K phosphorylation (Gao et al., 2021). Many studies have explored plant secondary metabolites as potent anticancer agents, and future perspectives have indicated that terpenes could be used to treat cancer owing to their vast biological properties.

#### 4.2.4 Sensitization of cancer cells through combination therapies

Cell sensitization often refers to the conditions under which cells respond to a stimulus in a sensitive manner. When talking about cancer cells, some terpene derivatives have been reported to possess a sensitizing effect on cancer cells *via* chemotherapies. A previous study reported the combination of doxorubicin (DOX) and β-elemene in MCF-7 breast cancer cells, where DOX-associated mean fluorescence intensity increased in the presence of β-elemene, indicating the accumulation of DOX in resistant cancer cells (Leary et al., 2018). β-elemene was also found to inhibit DNA repair in cisplatin-resistant human ovarian cancer cells by blocking cisplatin-mediated P13K/JNN and P13K/Akt activation (Figure 5). Moreover, β-elemene was found to increase the accumulation of doxorubicin and Rh123 as it inhibited the P-gp effect and downregulated the overexpression of E3 ubiquitin ligases in human gastric adenocarcinoma cells (Li et al., 2013). Other naturally bioactive monoterpenes also contribute to the sensitization of cancer cells, such as auraptene, which was found to reduce tumor markers in colon cancer cells and affect the size and integrity of tumors, resulting in a total reduction of 40% in colon spheres of HT-29 FOLFOX-resistant cells (Tayarani-Najaran et al., 2021). In addition to the sensitization of cancer cells, some terpenes have been demonstrated to act as radiosensitizers, such as perillic acid, which acts as a radiosensitizer in chemoradiation therapies for head and neck cancer. However, detailed mechanisms remain unexplored (Gong et al., 2021). According to an *in vivo* study, thymoquinone, a commonly known terpene, inhibits the nephrotoxicity of ifosfamide, a known chemotherapeutic agent, while simultaneously enhancing its anticancer activity (Farooq et al., 2021). According to an alternative study on the anticancer effects of thymoquinone, thymoquinone sensitizes pancreatic cancer cells to gentamicin and oxaliplatin during chemotherapy *via* the downregulation of transcription factors, NF-κB and Bcl-2 genes, and NF-κB-dependent anti-apoptotic genes (Gomathinayagam et al., 2020). Furthermore, artesunate a derivative of sesquiterpene lactone artemisinin was investigated for its sensitization effects which led to a conclusion that it could successfully sensitize castrate-resistant prostate cancer cells *via* suppressing NF-κB signaling apart from this, it was also found that artesunate has the potential to sensitize cells to doxorubicin leading to apoptotic cell death (Xu et al., 2020). Some diterpenoids also exhibit sensitization effects, such as in triptolide-sensitized androgen-sensitive prostate cancer cells. Further research concluded that they reduced the transcriptional activity of androgen receptors by inhibiting the expression of Sp1 and HSP70 (Kamran et al., 2022). Additionally, it sensitizes pancreatic cancer cells to gemcitabine through mitochondria-induced apoptosis and HSP27 inhibition (Guo et al., 2015). These studies suggest the phenomenal use of terpenes as anticancer agents through different mechanisms, which could lead to natural and non-toxic alternatives in the future.

### 4.3 Terpenes as antiviral agents

Worldwide health concerns have been heightened by the emergence of human viral diseases, which are characterized by their widespread occurrence, significant morbidity and mortality, and the risk of triggering outbreaks and pandemics. These illnesses stem from various viral sources, including RNA-based pathogens like coronaviruses and influenza viruses and DNA-based agents such as herpesviruses and poxviruses. The clinical presentations of human viral diseases span a broad spectrum, encompassing mild influenza-like symptoms to severe respiratory distress and neurological complications that may result in premature death (Mahmoudieh et al., 2024). The pharmacological potential of terpenes, emphasizing their role as promising SARS-CoV-2 inhibitors. It reviews their biological activities, mechanisms of action, and potential use as COVID-19 vaccine adjuvants. Safety and toxicity data, clinical trials, marketed products, and patents are also compiled. The findings underscore the importance of prioritizing phytochemical-based therapeutics and phytopharmaceutical formulations for COVID-19 treatment and prevention, even during interepidemic periods (Ceylan et al., 2025).

### 4.4 Terpenes as anti-inflammatory agents

Two novel compounds, a monoterpene (1) and a sesquiterpene (2), along with eleven known compounds, were isolated from *Platycladus orientalis*. Structural elucidation was performed using spectroscopic analysis and ECD calculations. Compounds 5–6, 9, and 12 demonstrated NLRP3-inflammasome inhibitory activity, indicating potential anti-inflammatory properties (Xie et al., 2025). According to Si-Yang Fang, study identified nine new glycosides (five terpene and four lignan) and thirteen known compounds in *Cyclocarya paliurus* leaves. Structures were determined using spectroscopic and computational methods. Compound 12 showed significant inhibition of LPS-induced nitric oxide production in RAW 264.7 cells, with efficacy comparable to dexamethasone. These findings suggest potential anti-inflammatory properties of the compounds, highlighting Cyclocarya paliurus as a promising source of bioactive natural substances for further research (Fang et al., 2025). The isolation of six new sesquiterpenes (magnogranoides A–F) and 10 known terpenes from Magnolia grandiflora L. leaves. Additionally, six new sesquiterpene derivatives were synthesized. The structures were identified using spectroscopic data and quantum chemical calculations. Notably, compound 1 has a novel 8/5/5 tricyclic ring structure, while compound 2 is the first 13-norguaiane-type sesquiterpenoid with a 5/7/5 tricyclic ring system. Four compounds (3, 7, 8, and 16) showed superior anti-neuroinflammatory activities in BV-2 cells compared to the positive control, minocycline (Wu et al., 2025). Terpenes as potential neuroprotective agents for Alzheimer’s disease (AD) treatment. It examines how terpenes may address multiple clinical indications of AD, including amyloid-β plaques, tau tangles, neuroinflammation, and oxidative stress (Lima and Medeiros, 2024).

## 5 Toxicity of terpenes

Certain terpenes, including β-caryophyllene, have cytoprotective, anti-inflammatory, and antioxidant properties, but most terpenes, especially monoterpenes, have a high potential for cytotoxicity, as demonstrated in a variety of model organisms. Linalool, humulene, α-terpineol, and terpinolene are among the most poisonous terpenes. Research on α-terpinene has shown that terpenes have harmful effects on pregnant rats (Araujo et al., 1996). Souza et al., who evaluated the monoterpene’s genotoxic effects in human cell cultures, HepG2, and leukocytes (Souza et al., 2020), provided new, significant information regarding citral toxicity. Therefore, before application, each terpene, which is frequently employed as a food additive and/or in medicinal applications, should be examined to determine whether it has any potential acute or long-term negative effects. According to a study, geraniol and citronellol were the most harmful to adults when applied topically and by immersion, respectively (Dambolena et al., 2016). As a result, in preclinical animal models, many terpenoids have demonstrated both anticancer and cancer prevention effects, in addition to cytotoxicity against a range of tumor cells. Terpene-derived essential oils cause cytotoxicity, harm organelles and cellular membranes, pro-oxidize proteins and DNA, and generate reactive oxygen species (ROS) (Srivastava and Singh, 2019). According to Castro et al. (Ashour et al., n. d.), the biological activity of EOs and their constituents on pest insects includes lethal toxicity by contact, fumigant toxicity, knockdown action, and behavior and feeding deterrence effects. Certain monoterpenes have harmful effects on the liver and other organs in the human body. High quantities of these compounds are hazardous to humans, harming the central nervous system, liver, kidneys, and lungs. Acute liver injury, including substantial increases in blood liver enzymes, prothrombin time lengthening, and hepatic encephalopathy (Bourgaud et al., 2001; Lassila et al., 2016), was observed in several patients after a single high dose.

## 6 Future perspective

Miscellaneous evidence indicates that green plants have long been a primary source of bioactive compounds as well as a major source of crude drugs used to cure a variety of human ailments. These plant metabolites serve as precursors for several pharmacological activities, including growth and maturation, signaling, catalytic activities, defence, reproduction, and interactions with other organisms. Plant growth and maturation are vital processes involving the production of primary metabolites, which are responsible for a variety of metabolic processes such as photosynthesis, respiration, and the formation of essential macromolecules. The end products of these primary metabolites are secondary metabolites (SMs), which occur in lesser quantities than primary metabolites. As they do not play a direct role in major plant development processes and are known to have impactful activities such as attracting pollinators or seed dispersal agents, controlling abiotic stress, utilization as industrial additives, and many pharmacological, physiological, and ecological effects, studies on these molecules have increased over the last 50 years [155]. This review emphasizes the use of natural terpenes as drug molecules against microbial infections and cancer that simultaneously possess low toxicity and high efficacy. As many studies have focused on the antimicrobial and anticancer properties of terpenes, this review presents a systematic approach towards defining the antimicrobial and anticancer properties of terpenes, as well as the basic nature, synthesis, and classes of terpenes, owing to their tremendous biological properties, thereby providing reliable data regarding the design of natural drugs against pathogenic microbial infections and cancer. Terpenes, the most extensive and versatile class of secondary metabolites (SMs), fulfill specialized chemical roles in plants and protect them from both abiotic and biotic stressors. Terpenes and their derivatives exhibit considerable potential as effective therapeutic agents with minimal adverse effects in the treatment of cancer and pathogens. The diverse nature and specialized functions of these individuals make them suitable candidates for innovative therapeutic approaches.

Although the potential therapeutic benefits of terpenes have sparked growing scientific interest, the journey from natural remedies to clinically validated treatments remains incomplete. A common theme in the current literature is that while many terpenes, such as limonene, β-caryophyllene, α-pinene, and farnesol, show a broad range of bioactivities *in vitro*, these results are not consistent with strong *in vivo* evidence. For example, the anti-inflammatory and anticancer effects observed in cell-based assays often lack thorough follow-up studies in animal models that investigate pharmacodynamics, toxicity profiles, or dosage optimizations. Moreover, there is a lack of advancements in clinical research. Only a few terpenes have been tested in humans, and most of the existing trials are preliminary, underpowered, or concentrated on formulations rather than the isolated bioactive compounds themselves. This highlights a significant gap: the absence of translational research connecting laboratory findings with therapeutic applications. Additionally, issues such as poor water solubility, instability, and low bioavailability continue to impede its clinical use, emphasizing the urgent need for innovative delivery systems (e.g., nanoemulsions and liposomes) and formulation strategies.

In conclusion, while the pharmacological potential of terpenes is promising, achieving their full therapeutic impact requires a shift from observational studies to targeted hypothesis-driven research. An integrative approach combining phytochemistry, pharmacology, and clinical sciences is crucial to transform these ancient plant molecules into modern medicines.

## Abbreviations

DMAPP, Dimethylallyl diphosphate; GPP, Geranyl pyrophosphate; IPP, Isopentenyl diphosphate; MEP, 2C-methyl-D-erythritol-4-phosphate; MVA, Mevalonate; SMs, Secondary metabolites; STS, Sesquiterpene synthase.
